# Overweight and Class I Obesity Are Associated with Lower 10-Year Risk of Mortality in Brazilian Older Adults: The Bambuí Cohort Study of Ageing

**DOI:** 10.1371/journal.pone.0052111

**Published:** 2012-12-14

**Authors:** Alline M. Beleigoli, Eric Boersma, Maria de Fátima H. Diniz, Maria Fernanda Lima-Costa, Antonio L. Ribeiro

**Affiliations:** 1 Faculdade de Medicina, Universidade Federal de Minas Gerais, Belo Horizonte, Brazil; 2 Department of Cardiology, Erasmus Medical Center, Rotterdam, The Netherlands; 3 Hospital das Clínicas, Universidade Federal de Minas Gerais, Belo Horizonte, Brazil; 4 Centro de Pesquisas René Rachou, Fundação Oswaldo Cruz, Belo Horizonte, Brazil; Wayne State University School of Medicine, United States of America

## Abstract

**Background:**

Prospective studies mostly with European and North-American populations have shown inconsistent results regarding the association of overweight/obesity and mortality in older adults. Our aim was **t**o investigate the relationship between overweight/ obesity and mortality in an elderly Brazilian population.

**Methods and Findings:**

Participants were 1,450 (90.2% from total) individuals aged 60 years and over from the community-based Bambuí (Brazil) Cohort Study of Ageing. From 1997 to 2007, 521 participants died and 89 were lost, leading to 12,905 person-years of observation. Body mass index (BMI) and waist circumference (WC) were assessed at baseline and at the 3rd and 5th years of follow-up. Multiple imputation was performed to deal with missing values. Hazard ratios (HR) of mortality for BMI or WC alone (continuous and categorical), and BMI and WC together (continuous) were estimated by extended Cox regression models, which were fitted for clinical, socioeconomic and behavioral confounders. Adjusted absolute rates of death at 10-year follow-up were estimated for the participants with complete data at baseline. Continuous BMI (HR 0.85; 95% CI 0.80–0.90) was inversely related to mortality, even after exclusion of smokers (HR 0.85; 0.80–0.90), and participants who had weight variation and died within the first 5 years of follow-up (HR 0.83; CI 95% 0.73–0.94). Overweight (BMI 25–30 kg/m^2^) was inversely (HR 0.76; 95%CI 0.61–0.93) and obesity (BMI ≥30 kg/m^2^; HR 0.85; 95% CI 0.64–1.14) not significantly associated with mortality. Subjects with BMI between 25–35 kg/m^2^ (23.8–25.9%) had the lowest absolute rates of death at 10-years follow-up. The association between WC and death was not significant, except after adjusting WC for BMI levels, when the relationship turned into marginally positive (HR 1.01; CI 95% 1.00–1.02).

**Conclusions:**

The usual BMI and WC cut-off points should not be used to guide public health and clinical weight control interventions in elderly in Brazil.

## Introduction

The public burden attributed to obesity relates to findings of increased morbidity and mortality associated with excessive weight in the general adult population [1]. Whether overweight and obesity lead to an increased risk of death in the elderly is still debatable. Several studies have shown decreased rates of death in older adults with excessive body weight in comparison to the ones with normal weight (18.50≤ BMI<25 Kg/m^2^) [2], [3], [4], [5], [6], [7]. This elderly “obesity paradox” is part of a broad range of originally unexpected findings of a protective effect of obesity on survival described in patients in dialysis [8], and with cardiovascular diseases [9],[10], [11],[12]. However, large epidemiological studies with predominantly Western European and North-American populations, found that despite a slight attenuation in the risk throughout the lifespan, overweight (body mass index [BMI] ≥25 Kg/m^2^) and obesity (BMI ≥30 Kg/m^2^) increase the risk of death in older adults [13], [14]. The controversial results on whether BMI levels associated with high risk of death in the general adult population apply to the elderly may be explained by particularities of each elderly population, such as ethnicity and lifestyle habits, differences in the methods used to assess body fat, and length of follow-up. Use of single point or longitudinal anthropometric measurements, and diverse approach to adjusting for confounders can also explain the different findings [15].

Studying this issue in other Western populations of older adults in the context of demographic, nutritional and technological transitions, such as the Brazilian one [16], is critical to planning public health policies. These strategies may be of great impact due the combination of the ageing of the population and the increase in the prevalence of obesity. In Brazil, the number of individuals with 65 years or over increased 54% in the last 20 years, and the prevalence of overweight and obesity among them raised from 30% to 55%, and from 6% to 17%, respectively [17], [18]. Additionally, this investigation may aid to clarify the role of individual clinical interventions towards overweight and obesity.

The objective of this study was to investigate the relationships between overweight and obesity assessed by BMI and waist circumference (WC) at various time-points, and the overall mortality in 10 years of follow-up in the community-dwelling elderly of the Bambuí (Brazil) Cohort Study of Aging (BHAS). Additionally, we aimed at verifying whether these relationships were modified by age, gender, smoking and physical activity, and in subgroups of non-smokers, and participants who had stable weight and survived the first five years of follow-up.

## Methods

### Ethics Statement

Participants signed an informed consent form and authorized death certificate verification. The BHAS was approved by the ethics board of the Fundação Oswaldo Cruz, Belo Horizonte, Brazil.

### Study design and population

The study was conducted in the Bambuí City (approximately 15,000 inhabitants), located in the state of Minas Gerais in the southeast region of Brazil. Procedures used in the BHAS have been described in detail elsewhere [19]. Briefly, the baseline cohort population comprised 1,606 (92.2%) of all the 1,742 residents aged 60 years or more on January 1^st^, 1997, who were identified by means of a complete census carried out in the city. Baseline data collection was performed from February to May 1997, comprising standardized interviews, blood tests, blood pressure measurements, and electrocardiograms (ECGs). Bambuí city was an endemic area of Chagas disease (*Trypanossoma cruzi* infection) and the infection remained highly prevalent in old persons due to a cohort effect, despite the successful interruption of the transmission by 1970.

### Outcome Ascertainment

Deaths assigned to any cause occurring from study enrolment to December 31^st^, 2007 were included in this analysis. Deaths reported by next of kin during the annual follow-up interview were ascertained through the Brazilian mortality information system (Sistema de Informações sobre Mortalidade) with the permission of the Ministry of Health. Death certificates were obtained for 98.9% of all deceased participants.

### Anthropometric measurements

Two high-precision digital scales (range 0–150 kg×0.1 kg) were used for the measurement of weight (kg) and ACMS Portable Stadiometer kit (CMS Weighing Equipment Ltd., London) was used for measurements of height (cm). WC was measured at umbilicus height using inelastic tapes. The reliability of these measurements was determined by repeating them in a 5% cohort of all of the study participants. All measures were performed with individuals wearing light clothing and no shoes in the baseline and repeated in the participants who survived in 2000 and 2002 [20]. BMI was calculated using the conventional formula of weight in kilograms divided by the square of the height in meters, and categorized according to the World Health Association (WHO) convention: underweight (<18.5 Kg/m^2^); normal weight (18.5–24.9 Kg/m^2^); overweight (25–29.9 Kg/m^2^); obesity (≥30 Kg/m^2^). WC was classified according to WHO criteria into normal (<88 cm in women and <102 cm in men) or increased (≥88 cm in women and ≥102 cm in men) [21].

### Other measures

Systolic blood pressure was defined as the mean of the two lowest measurements out of three using standard protocols. The use of anti-hypertensive medication and digoxin was assessed by standardized interview. Fasting blood glucose and creatinine levels were measured by traditional enzymatic methods. Diagnosis of chronic Chagas disease was defined in the baseline by seropositivity of three different assays performed concurrently as detailed elsewhere [19]. Plasmatic B-type natriuretic peptide (BNP) was measured as described elsewhere and is a sensitive marker of heart dysfunction and a strong predictor of mortality in BHAS [22]. Diabetes was defined as a 12-h-fast glucose ≥126 mg/dL and/or the use of insulin or oral hypoglycaemic agents. 12-lead ECGs were digitally recorded at rest using standardized procedures, analyzed by experienced cardiologists at the ECG Reading Center (EPICARE Center, Wake Forest University School of Medicine, Winston-Salem, NC), codified according to the Minnesota Code (MC) [23], and classified as abnormal in the presence of major abnormalities [24] or of frequent supra-ventricular and ventricular premature beats (MC 8.1.1, 8.1.2 or 8.1.3). Monthly household income, education, current smoking and leisure physical activity (walking and/or practicing any other physical exercise for at least 20–30 min within the last 90 days) were verified through standardized interview [19].

### Statistical analysis

We used histograms and Shapiro-Wilk tests to verify normality. Skewed variables were log-transformed. The Chi-square test for linear trends, the ANOVA and the Kruskal-Wallis tests were used, respectively, to verify differences in frequencies, mean and median for the categorical and continuous baseline variables across BMI categories. We use the ANOVA test for repeated measures with Greenhouse-Geisser correction and the Friedman test to compare the mean and median values, respectively, of BMI and WC in 1997, 2000 and 2002. Spearman coefficients were used to calculate the correlation between BMI and WC at each time-point.

To deal with missing values, we performed missing value analysis. After assuming that these values were missing at random and performing log-transformations for variables with skewed distribution, we performed multiple imputation [25], [26]. This procedure consisted in generating five complete datasets by a set of values yielded by logistic and linear regression models, for categorical and continuous variables, respectively. Both the predictors and the outcome were used to fit these models, as well as other variables of the Bambuí dataset which were thought to be related to the missing values, but were not directly implicated in the present analysis. The combination of these datasets was used to calculate hazard ratios (HR) and 95% confidence intervals (CI) of mortality for BMI alone (continuous plus quadratic term, and categorical), WC alone (continuous and categorical), and BMI (continuous plus quadratic term) plus WC (continuous). Extended Cox regression models were chosen to test these associations because the measurements of BMI and WC at baseline, 3^rd^ and 5^th^ years of follow-up were used as time-dependent covariates, which by definition do not satisfy the proportional hazard assumption [27]. Each model was adjusted for both an extensive and a restricted set of co-variables. The extensive model included age (continuous), gender, Chagas disease (no, yes), current smoking (no, yes), log-transformed serum B-type natriuretic peptide (continuous), the presence of major ECG abnormalities (no, yes), systolic blood pressure (continuous), total cholesterol (continuous), diabetes mellitus (no, yes), log-transformed serum creatinine levels (continuous), physical activity (< or ≥3–5 times/week), education (never, <4, ≥4 school years), monthly household income (1–4, 4–10, ≥10 minimum wage), anti-hypertensive medication (no, yes) as co-variables. In the restricted model, we excluded total cholesterol, diabetes, systolic blood pressure and anti-hypertensive treatment, in order to verify whether the presence of these intermediates on the causal pathway between obesity and mortality led to an inappropriate attenuation of the risk of mortality associated with an excess of body fat. Results yielded by multiple imputation datasets were compared to the ones with complete data.

Additionally, we added multiplicative interaction terms between BMI/ WC, and age, gender, smoking and physical activity, and performed sensitivity analyses by investigating the relationships of interest in the subgroups of non-smokers, and of participants who did not lose (≥10% decrease) or gain (≥10% increase) weight significantly and survived the first five years of follow-up. The absolute rate of events in the 10-years follow-up was calculated according to each BMI unit and also to BMI WHO categories for the whole population and for the subgroups of interest by multiplying the exp (absolute risk) by the baseline survival function, which was estimated by Kaplan–Meier curves. All analyses were performed considering p<0.05 as a significant level. We used SPSS 17.0 for Windows (Chicago, IL), and SAS 9.2 for Windows (Institute Inc. Cary, NC, USA).

## Results

Of the 1606 cohort subjects enrolled, 1,450 had BMI measures at baseline and 89 (5.5%) were lost to follow-up. Those who were followed were older (68.9 years (SD 7.2) versus 67.3 years (SD 5.9), respectively) and had higher BMI values at baseline (24.8 kg/m^2^, IQR 21.7–27.9, versus 23.4 kg/m^2^, IQR 21.7–28.0; p = 0.047) than those who were lost to follow-up. There were 12,905 person-years of observation and 521 (35.9%) deaths in a mean follow-up time of 8.9 (SD 3.1) years. Overall, 12.9% of values of all variables and cases had missing values, were imputed and used for estimation of HR. Regarding the repeated anthropometric measures, participants who survived had the following number of missing values in 2000 and 2002, respectively: 153(12.0%) and 167 (14.2%) missing weight; 160 (12.4%) and 172 (14.6%) missing height; 160 (12.4%) and 172 (14.6%) missing WC values. Median BMI was 24.8 kg/m^2^ (IQR 21.6–28.0), 24.7 kg/m^2^ (IQR 21.8–27.8), 24.6 kg/m^2^ (IQR 21.6–27.9); p<0.001, and mean WC was 91 cm (SD 11.2), 90 cm (SD 11.0), 93 cm (SD 12.9); p<0.001, at baseline, 2000 and 2002, respectively. BMI and WC were moderately to strongly correlated (r = 0.82, r = 0.81 and r = 0.66, at baseline, 3^rd^ and 5^th^ years of follow-up, respectively). The number of subjects with waist increase was significantly different across BMI categories at baseline: one in the underweight (0.0%), 130 (20.2%) in the normal weight, 328 (63.8%) and 178 (94.2%) in the obese category (p<0.001). The baseline characteristics of all the eligible subjects and according to BMI categories are depicted in Table 1.

**Table 1:** Characteristics of participants with Body Mass Index (BMI) measured at baseline, and comparison according to BMI category.

		BMI category				*
Characteristics	Total	Underweight	Normal weight	Overweight	Obesity	p value
	(n = 1450)	(n = 104;7.2%)	(n = 643;44.3%)	(n = 514;35.4%)	(n = 189;13.0%)	
Deaths, n (%)	521	58	262	145	56	0.01
	(35.9)	(58.6)	(41.5)	(28.8)	(30.3)	
Age, mean (SD)	68.9	72	69	68	67	<0.001
	(7.0)	(7.0)	(7.0)	(7.0)	(6.0)	
Female sex, n (%)	879	58	337	330	154	<0.001
	(60.9)	(55.8)	(52.4)	(64.2)	(81.5)	
Smoking, n (%)	251	33	150	55	13	<0.001
	(17.7)	(33.3)	(23.8)	(10.9)	(7.0)	
BNP in pg/ml, median (IQR)	80	102	87	74	65	0.010
	(43-148)	(56-163)	(49–160)	(40–136)	(35–131)	
Chagas disease, n (%)	536	57	263	155	61	<0.001
	(37.8)	(57.6)	(41.7)	(30.8)	(33.0)	
Systolic blood pressure in mm Hg, mean (SD)	137	130	137	139	140	0.002
	(22.4)	(24.0)	(24.0)	(21.0)	(21.0)	
Diabetes mellitus, n (%)	207	6	63	83	55	<0.001
	(14.6)	(6.1)	(10.0)	(16.5)	(29.7)	
Serum creatinine in mg/dL, median (IQR)	0.85	0.90	0.86	0.85	0.83	0.070
	(0.77–0.99)	(0.77–1.01)	(0.76–0.99)	(0.75–0.98)	(0.71–0.98)	
Total cholesterol, mean (SD)	234	231	239	235	236	0.008
	(49.2)	(49.0)	(50.0)	(49.0)	(48.0)	
ECG with major abnormalities†	574	49	279	179	67	0.001
	(40.5)	(49.5)	(44.2)	(35.5)	(36.2)	
Physically active‡, n (%)	312	10	123	139	40	0.002
	(21.5)	(9.6)	(19.1)	(27.1)	(21.2)	
Education§, n (%)						
Lower	454	47	236	125	46	
	(31.3)	(45.2)	(36.7)	(24.3)	(24.3)	
Intermediate	837	54	354	321	108	
	(57.7)	(51.9)	(55.1)	(62.5)	(57.1)	
Higher	159	3	53	68	35	<0.001
	(11.0)	(2.9)	(8.2)	(13.2)	(18.5)	
Monthly household income ||, n (%)						
Lower	962	79	459	310	114	
	(66.9)	(76.7)	(71.8)	(61.0)	(60.3)	
Intermediate	358	23	136	148	51	
	(24.9)	(22.3)	(21.3)	(29.1)	(27.0)	<0.001
Higher	119	1	44	50	24	
	(8.3)	(1.0)	(6.9)	(9.8)	(12.7)	

* P value: Students t test, Pearson's chi-square test for trends and the Kruskal Wallis test for differences between means, frequencies and medians, respectively †Major ECG abnormalities were defined by the following alterations and Minnesota codes (MC): ventricular conduction defect (MC 7.1, 7.2, 7.4, or 7.8); possible old myocardial infarction (MC1.1.x, 1.2.x and 1.3.x and (4.1.x, 4·2, 5·1, or 5·2)), major isolated ST segment and T wave abnormalities (MC 4.1.x, 4.2, 5.1, or 5.2), left ventricular hypertrophy (MC 3.1 and (4.1.x, 4.2, 5.1, or 5.2)), atrial fibrillation (MC 8.3.1, 8.3.3), major atrio-ventricular conduction abnormalities (MC 6.1.1, 6.2.1, 6.2.2, 6.2.3, 6.4.1, 8.6.1, 8.6.2), pacemaker use (MC 6.8.1), other major arrhythmias (MC 8.2.1, 8.2.2, 8.2.3, 8.2.4, 8.3.2, 8.3.4, 8.4.·2), frequent supraventricular and ventricular premature beats (MC 8.1.1, 8.1.2 or 8.1.3) ‡ Leisure physical activity (walking or any other physical exercise) for at least 20–30 min, ≥3–5 times/week § Education: lower category-never studied, intermediate category −<4 school years, higher category −≥4 school years) || Monthly household income in minimum wages (lower category 1–4, intermediate category 4–10, higher category ≥10).

**Figure 1:**
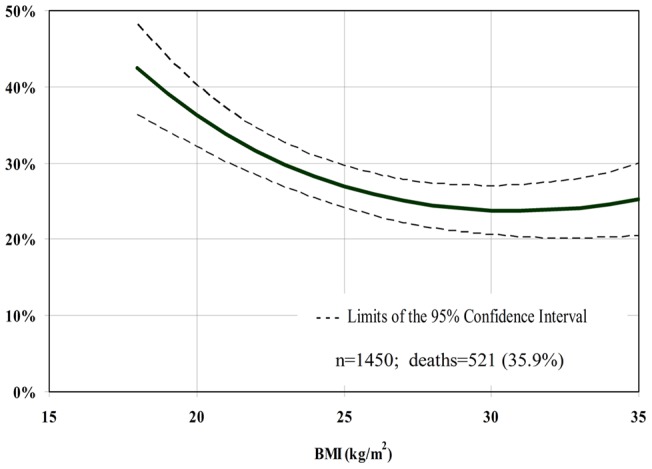
10-year cumulative incidence of death per BMI unit at baseline.

**Figure 2:**
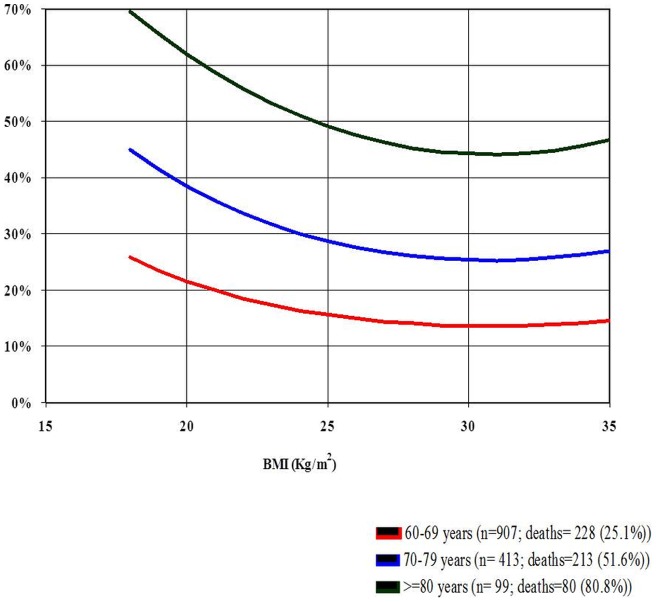
10-year cumulative incidence of death per BMI unit at baseline according to age groups.

**Figure 3:**
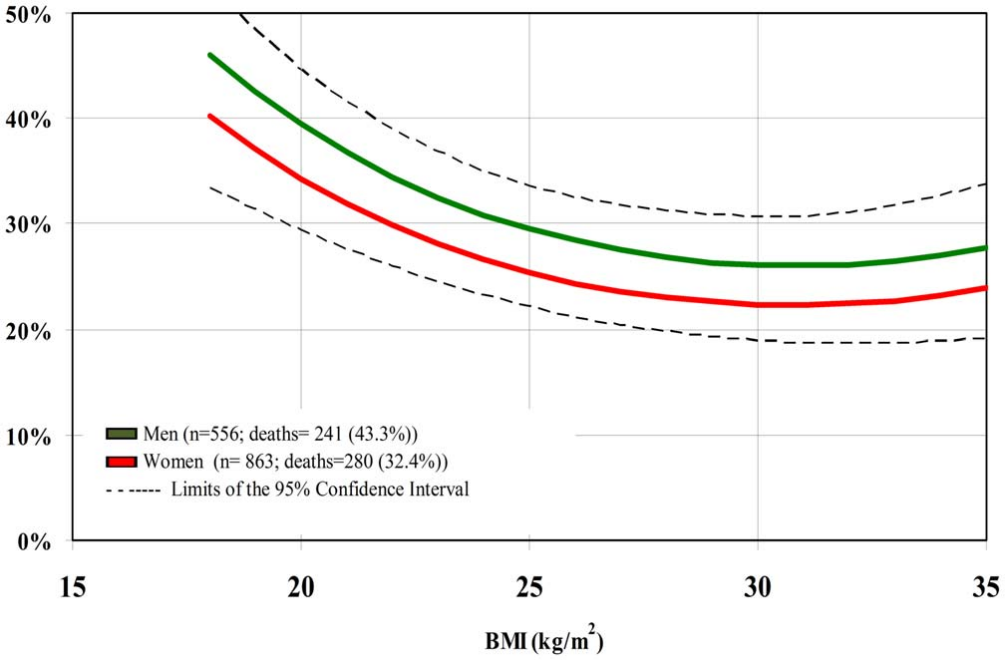
10-year cumulative incidence of death per BMI unit at baseline according to gender.

**Figure 4:**
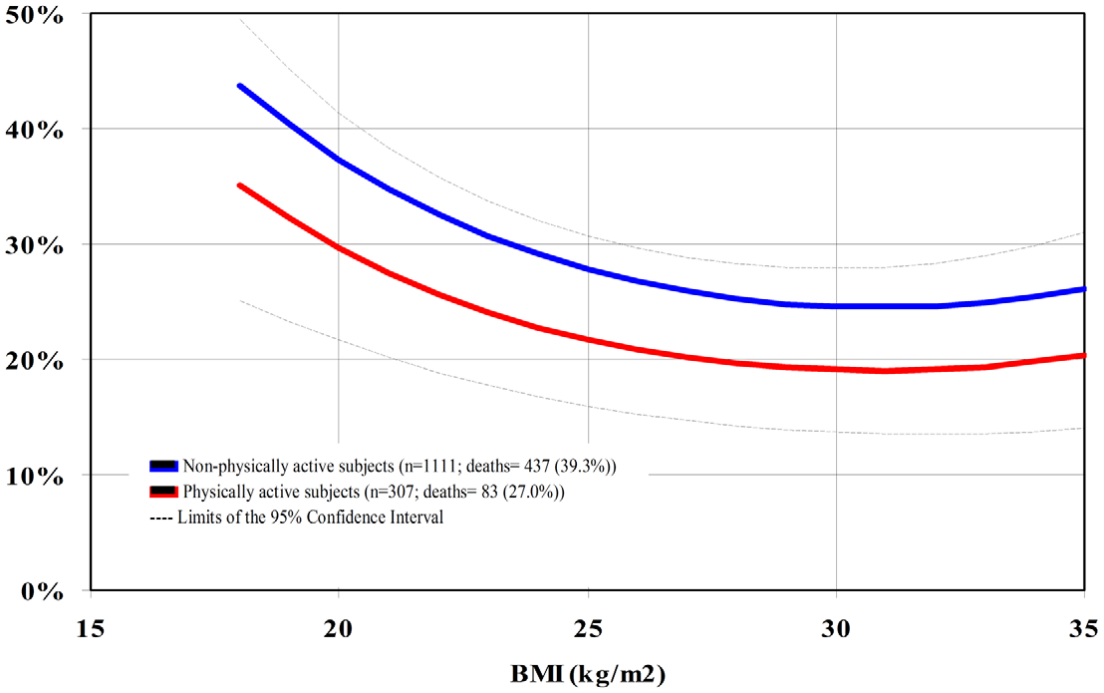
10-year cumulative incidence of death per BMI unit at baseline according to physical activity status.

For BMI as a continuous variable, we found a significant inverse relationship with mortality. The magnitude of HR did not differ when the models were performed with all the participants and with the subgroups of interest. Neither did the results change appreciably between the extensive and restricted set of co-variables (Tables 2, 3, and 4). Thus, we considered the extensive models for the whole population for estimating the 10-year cumulative incidence of death. Across the observed BMI values in the whole population, BMI = 30 Kg/m^2^ (23.8%) and the range between 25–35 Kg/m^2^ (23.8–25.9%) at baseline were associated with the lowest absolute rates of death at 10-year follow-up (Figure 1).Age, gender (p for interaction = 0.56 and 0.83, respectively; Figures 2 and 3) and smoking status (p for interaction = 0.85) did not modify the association between BMI and mortality. Physical activity modified the association between BMI and death (p for interaction = 0.04), and the 10-year absolute rate was lowest at BMI 32 kg/m^2^ for the physically active (19.1%) and at BMI 30 kg/ m^2^ (24.6%) for the non-physically active (Figure 4).

**Table 2:** Hazard ratios (HR) and 95% confidence intervals (95% CI) of mortality related to body mass index (BMI) and waist circumference (WC) for all participants.

Models	HR (95% CI) Multiple Imputation dataset		HR (95% CI) Complete dataset	
	BMI	WC	BMI	WC
Unadjusted	0.78 §	0.99 ^||^	0.77 §	0.99 ^||^
	(0.74–0.82)	(0.98–1.00)	(0.72–0.83)	(0.98–1.00)
Restricted model*	0.86 §	1.00	0.85 §	1.00
	(0.81–0.91)	(0.99–1.01)	(0.79–0.92)	(0.99–1.01)
Extensive model†	0.85 §	1.00	0.85 §	1.00 ^||^
	(0.80–0.90 )	(0.99–1.00)	(0.79–0.92)	(1.00–1.02)
BMI and WC together‡	0.81 §	1.01 ^||^	0.81 §	1.00
	(0.76–0. 86)	(1.00–1.02)	(0.73–0.89)	(1.00–1.03)

* Restricted model: Time-dependent BMI (continuous and quadratic term) or WC (continuous) plus age, sex, smoking (no, yes), Chagas disease (no, yes), log-transformed creatinine (continuous), log-transformed BNP levels (continuous), major ECG abnormalities (no, yes), physical activity within the last 90 days (no, yes), household income (<4, 4–10, ≥10 minimum wage), and education (never, 1–4, ≥4 years).

† Extensive model: Restricted model plus diabetes (no, yes), total cholesterol (continuous), systolic blood pressure (continuous), anti-hypertensive treatment (no, yes).

‡ BMI and WC together: BMI (continuous and quadratic term), WC (continuous) plus extensive model.

§ p<0.001; ^||^ p<0.05; all other values p>0.05.

**Table 3:** Hazard ratios (HR) and 95% confidence intervals (95% CI) of mortality related to body mass index (BMI) and waist circumference (WC) for non-smokers.

Models	HR (95% CI) Multiple Imputation dataset		HR (95% CI) Complete dataset	
	BMI	WC	BMI	WC
Unadjusted	0.79 §	0.99 ^||^	0.77 §	0.99
	(0.75–0. 83)	(0.98–1.00)	(0.71–0.83)	(0.98–1.00)
Restricted model	0.86 §	1.00	0.85 §	1.00
	(0.81–0.91)	(0.99–1.01)	(0.78–0.92)	(0.99–1.01)
Extensive model	0. 85 §	1.00	0.84 §	1.00
	(0.80–0. 90)	(0.99–1.00)	(0.77–0.91)	(0.99–1.01)
BMI and WC together	0. 82 §	1.01	0.82 §	1.00

* Restricted model: Time-dependent BMI (continuous and quadratic term) or WC (continuous) plus age, sex, smoking (no, yes), Chagas disease (no, yes), log-transformed creatinine (continuous), log-transformed BNP levels (continuous), major ECG abnormalities (no, yes), physical activity within the last 90 days (no, yes), household income (<4, 4–10, ≥10 minimum wage), and education (never, 1–4, ≥4 years).

† Extensive model: Restricted model plus diabetes (no, yes), total cholesterol (continuous), systolic blood pressure (continuous), anti-hypertensive treatment (no, yes).

‡ BMI and WC together: BMI (continuous and quadratic term), WC (continuous) plus extensive model.

§ p<0.001; ||p<0.05; all other values p>0.05.

**Table 4:** Hazard ratios (HR) and 95% confidence intervals (95% CI) of mortality related to body mass index (BMI) and waist circumference (WC) for participants who survived the first 5 years of follow-up and maintained stable weight.

Models	HR (95% CI) Multiple Imputation dataset		HR (95% CI) Complete dataset	
	BMI	WC	BMI	WC
Unadjusted	0.75 §	0.99 ^||^	0.85 §	1.00
	(0.67–0.84)	(0.98–1.00)	(0.79–0.91)	(0.99–1.01)
Restricted model	0.82 §	1.00	0.85 §	0.99
	(0.73–0.93)	(0.99–1.01)	(0.79–0.91)	(0.98–1.01)
Extensive model	0.83 §	1.00	0.82 §	1.00
	(0.73–0.94)	(0.99–1.01)	(0.74–0.90)	(0.99–1.01)
BMI and WC together	0. 80 §	1.01	0.81 §	1.01
	(0.71–0.90)	(1.00–1.03)	(0.72–0.91)	−1.03)

* Restricted model: Time-dependent BMI (continuous and quadratic term) or WC (continuous) plus age, sex, smoking (no, yes), Chagas disease (no, yes), log-transformed creatinine (continuous), log-transformed BNP levels (continuous), major ECG abnormalities (no, yes), physical activity within the last 90 days (no, yes), household income (<4, 4–10, ≥10 minimum wage), and education (never, 1–4, ≥4 years).

† Extensive model: Restricted model plus diabetes (no, yes), total cholesterol (continuous), systolic blood pressure (continuous), anti-hypertensive treatment (no, yes).

‡ BMI and WC together: BMI (continuous and quadratic term), WC (continuous) plus extensive model.

§ p<0.001; ||p<0.05; all other values p>0.05.

In the analysis based on current BMI criteria defined by WHO, we found that compared to normal weight, underweight subjects had a higher (HR 1.54; 95% CI 1.02–1.89), and overweight (HR 0.76; 95% CI 0.61–0.93) had a lower relative risk of dying at 10-year follow-up. Obesity was not significantly associated with death (HR 0.85; 95% CI 0.64–1.14). Absolute rates of death at 10-year follow-up were lower in the overweight (24–27%; 95% CI 20 to 30%) and obese (24–25%; 95% CI 20 to 30%) categories, and higher in the underweight group (39–42%; 95% CI 34 to 48%) than in the normal weight category (27–36%; 95% CI 24 to 40%).

Neither was the relationship between WC alone (continuous) and mortality significant in the whole population nor in the subgroups of interest (Tables 2 to 4). This relationship was not modified by age, gender, smoking status or physical activity (p for interaction = 0.63, 0.69, 0.87 and 0.09, respectively). After controlling for WC, BMI retained its protective effect on death, whereas after controlling for BMI, WC increased the risk of death in the whole population, but not in the subgroups of interest (Tables 2 to 4). When fully adjusted, waist increase (categorical) was not significantly associated with mortality (HR 0.89; 95% CI 0.73–1.08 in the whole population, HR 0.82; 95% CI 0.67–1.01 in the subgroup of non-smokers, and HR 0.81; 95% CI 0.54–1.20 in the subgroup of survivors without weight variation within the first five years of follow-up).

## Discussion

The present analysis based on anthropometry at various time-points found that BMI alone or adjusted for WC is inversely related to mortality in a population of Brazilian elderly. These results did not change appreciably when we tried to minimize potential bias by excluding smokers, and participants who had weight variation and died early. Additionally, we demonstrated that when BMI was assessed by WHO categories, overweight was inversely and obesity not significantly associated with death. When the adjusted absolute rates of death were taken into account, BMI levels within the range of overweight and class I obesity were associated with the lowest rates of death in the follow-up of 10 years of the elderly of the BHAS.

Although the findings of large epidemiological studies in North-America and Europe have shown an increase in the risk of death associated with BMI ≥25 kg/m^2^ in older adults [13], [14], [28], our results are in line with several others in the fields of geriatrics/gerontology and public health [2], [3], [5], [6], [7] which did not enroll elderly by the presence of chronic diseases. The interrelations between adiposity, fitness and mortality are among the possible methodological and biological factors explanations to these different findings regarding the “obesity paradox”. Studies which investigated the issue in patients with coronary heart disease (CHD), have demonstrated that fitness influences the occurrence of the “obesity paradox” [9], [29], [30]. Patients with CHD with high levels of fitness survived longer than the ones with low levels of fitness, independently of BMI category [9], [30], whereas overweight and obesity were protective factors in those with high fitness, but not in those with low fitness [29] in another study with middle-aged and older adults. The lack of data on fitness in the BHAS prevented us for adjusting for this potential confounder. However, we found that physical activity significantly modified the association between BMI and mortality. Although BMI values associated with the lowest rates of death were within the obesity range in both groups, the values were higher in the active than in the non-active group (32 versus 30 kg/m^2^). We do think that this statistical interaction implies biological synergy, not only because physical activity acts on intermediate mechanisms of death, such as oxidation, endothelial dysfunction, and insulin resistance [4], [31], but also because it may be a proxy of fitness [32] and other unmeasured confounding factors, such as healthy lifestyle behavior and better functional status.

Death of the individuals more susceptible to the adverse effects of obesity leading to selective survival bias, and weight loss associated with underlying disease leading to reverse causality bias may account for an underestimation of the effect of obesity on mortality and explain our results. Reverse causality was addressed in our study by excluding both subjects who had a significant weight change between the first and last measurement and died prematurely. Since both weight loss and gain have been demonstrated to be related to chronic disease and increased mortality [33], we excluded subjects with weight variation in either direction. However, we are aware that this approach might not have fully addressed the issue [34]. The methodological approach to smoking is another factor that is thought to influence on the estimations of the effect of obesity on mortality. Given that smoking is related to both low weight and mortality, the “obesity paradox” could be explained by the protective effect of high BMI levels in smokers rather than in non-smokers [35], [36]. However, this does not seem to be the case in our population as there was not a significant interaction between smoking and BMI or WC, and the protective effect of high BMI levels was consistent both after adjusting for current smoking, and after excluding smokers from the analysis.

Regarding the biological mechanisms to explain the “obesity paradox” in the elderly, a greater metabolic reserve and a potentially better nutritional status in overweight and obese subjects than in those with underweight or normal weight are possible mechanisms. Inflammatory and neuro-hormonal mechanisms, such as neutralization of the adverse effects of tumor necrosis factor (TNF)-alfa by soluble receptors in the adipose tissue [37], neutralization of lipopolysaccharides (stimulants of the release of inflammatory cytokines) by higher cholesterol levels in the plasma of obese, and protective alterations in the activation of neuro-hormonal pathways in the obese subjects [38], are commonly investigated to explain the “obesity paradox” in patients with cardiovascular disease (CVD). As most of these patients are elderly, it is possible that these factors may explain at some extent the existence of the “obesity paradox” in elderly not specifically enrolled by CVD as well.

An additional explanation to the findings of the “obesity paradox” mainly in the elderly and in different clinical/epidemiological settings involves the accuracy of the anthropometric measures as surrogates of adiposity. As a measure of total body mass, BMI reflects both lean and fat mass. The aging process involves decline in stature and changes in body composition, such as loss of lean and increase of fat depots, which may lead to either underestimation or overestimation of overall adiposity by BMI [39]. Normal BMI levels, thus, may reflect a combination of low muscle mass and high adiposity rather than normal adiposity, which can explain the lower survival rates of elderly within the normal BMI range, due to the well-established increased risk of disability associated with loss of muscle mass and/or function [40], [41]. Contrary to this hypothesis, however, are previous findings of a strong correlation between BMI and total body fat in the elderly [42], and of non-significant [32] and inverse association between body fat measured by more accurate methods and death in community-dwelling elderly [5]. These latter findings suggest that BMI might not only be a good marker of adiposity in the elderly, but also that adiposity *per se* might offer some protection against death. Lower rates of loss of bone mass, which may reduce the risk of falls and protect from trauma, and larger nutritional reserves in periods of acute stress [43] are among the potential advantages of higher amounts of body fat in older adults.

However, BMI does not reflect body fat distribution, which can be particularly important in the elderly due to body fat redistribution associated with ageing. In this regard, neither did we find a significant association between WC and death, nor between waist/ hips ratio and death (data not shown). This relationship was not modified by gender, age, physical activity and smoking status. Previous studies with older adults showed contradictory results [44], [45]. The increase in the risk of death associated with augmented WC levels is usually explained by the interrelations between WC, visceral fat, and conditions considered to be pro-atherogenic, such as insulin resistance, hypertriglyceridemia, high atherogenic cholesterol profile and inflammation [42], [46]. However, ethnicity and sex might change the magnitude of the correlation between WC and visceral fat [47]. We think that an attenuation of the association between WC and death might have happened in our population in case total body mass [48] and/or the abdominal subcutaneous component, which has a less pathogenic metabolic and cardiovascular profile [49], had been proportionately more important than visceral fat in determining WC values. A modest increase in the risk of death related to WC levels was observed when WC was adjusted for BMI in the whole population, but not in the subgroups of interest. These results are in line with studies with different populations of community-dwelling elderly [3], [45], [50], as well as in elderly patients enrolled by CHD [51]. It is argued that the adjustment of WC for BMI yields a less confounded estimate of the association between WC and mortality because under these circumstances BMI reflects better lean than fat mass [52]. This is thought to be particularly true in elderly with low BMI, but our study lacked power to detect differences in the influence of WC on mortality according to BMI categories because of both the small number of subjects in the underweight category with high WC, and of subjects in the obese category with low WC.

Our findings suggest that current BMI cut-off points established as of high risk for the adult population did not grade appropriately the risk of death in the elderly subjects of the BHAS. We believe that these results can be generalized to the Brazilian urban elderly population, due to similarities in terms of the predominance of the female gender, educational level, prevalence of overweight and obesity, tobacco use, and diabetes, but not physical activity [18]. Hence, public policies and individual recommendations of weight loss for elderly individuals with BMI ≥25 kg/m^2^ in Brazil based on the argument that lean people have longer survival do not seem adequate particularly if a proportionate reduction in WC does not accompany the reduction in BMI. However, intentional weight loss may lead to other benefits which were not evaluated in this investigation, such as the prevention of diabetes, disability and physical frailty, chronic knee and back pain [28], [40]. Thus, the premises of individualization of treatments and focus on quality of life rather than on the search for the ‘ideal BMI’ seem to be even more important in older adults. For the elderly who possibly benefit of weight loss, our findings have two main implications. First, the increased risk of death associated with WC when adjusted for BMI, suggests that interventions should be focused on reducing WC rather than BMI. Secondly, the enhanced protection of overweight/obese elderly by physical activity suggests that physical activity is a key element in the weight management in the elderly, corroborating the findings of some interventional studies [53], [54].

### Strengths and limitations

As far as we know, our study is unique in prospectively investigating the association of anthropometric measures and long-term mortality in a Brazilian elderly population, and provides further contribution to the field by the use of direct and multiple measurements of BMI and WC to investigate this relationship, and not only baseline self-reported data. The minimal number of losses to follow-up (6%), the high rate of objective outcome events (35.9%), and the sensitivity analyses make our results quite confident. Moreover, the large set of co-variables allowed us to account not only for biological/clinical factors, but also for potential socioeconomic and lifestyle-related determinants of death.

Several limitations warrant mention. The risk of death in severely obese elderly was not properly addressed in this study due to the low number of subjects (14; 0.9%) with BMI ≥40 kg/m^2^ at baseline. The generalizability of the results to the elderly male population is also limited because of the small number of cases of obesity (35; 5.5%) and abdominal obesity (73; 11.4%) among men in our population. Residual confounding is possible because of lack of data on fitness and on dietary patterns, and the quite simple assessment of physical activity. The results on the influence of smoking are weakened at some extent, due to lack of data on past habits and quantity of smoking. Moreover, further insights into the mechanisms of the “obesity paradox” are limited by the potential limitations of BMI as a surrogate of adiposity in the elderly, and by the absence of more accurate measurements of muscle mass/function, as well as of body composition and body fat distribution. It is noticeable, however, that techniques such as abdominal computed tomography or magnetic resonance imaging are expensive, difficult to use in large-scale epidemiological research and most importantly, are rarely available and not always adequate for clinical settings.

In conclusion, the inverse relationship between mortality and BMI measured at various time-points and the lack of significant association with WC alone in the 10-year follow-up of a Brazilian cohort of elderly suggest that the usual cut-off points used to grade the effect of overall and abdominal obesity on the risk of mortality in adults do not apply to the elderly. Hence, the strict use of these values to guide obesity treatment and define public policies in obesity in Brazil should be avoided. In addition, the increase in the risk of death when WC was adjusted for BMI, and the joint protective effects of BMI and physical activity, suggest that abdominal obesity and physical activity should probably be the focuses of interventions in the elderly who are thought to benefit of weight loss.
